# Expression profiling of lymph nodes in tuberculosis patients reveal inflammatory milieu at site of infection

**DOI:** 10.1038/srep15214

**Published:** 2015-10-15

**Authors:** Abhijit Maji, Richa Misra, Anupam Kumar Mondal, Dhirendra Kumar, Divya Bajaj, Anshika Singhal, Gunjan Arora, Asani Bhaduri, Andaleeb Sajid, Sugandha Bhatia, Sompal Singh, Harshvardhan Singh, Vivek Rao, Debasis Dash, E Baby Shalini, Joy Sarojini Michael, Anil Chaudhary, Rajesh S. Gokhale, Yogendra Singh

**Affiliations:** 1 CSIR-Institute of Genomics & Integrative Biology, Mall Road, Delhi, India; 2 Academy of Scientific & Innovative Research (AcSIR), CSIR-IGIB, Delhi, India; 3 Department of Zoology, University of Delhi, Delhi, India; 4 Hindu Rao Hospital, Malka Ganj, Delhi, India; 5 Christian Medical College, Vellore, Tamil Nadu, India; 6 Rajan Babu Institute of Pulmonary Medicine and Tuberculosis, Delhi, India

## Abstract

Extrapulmonary manifestations constitute 15 to 20% of tuberculosis cases, with lymph node tuberculosis (LNTB) as the most common form of infection. However, diagnosis and treatment advances are hindered by lack of understanding of LNTB biology. To identify host response, *Mycobacterium tuberculosis* infected lymph nodes from LNTB patients were studied by means of transcriptomics and quantitative proteomics analyses. The selected targets obtained by comparative analyses were validated by quantitative PCR and immunohistochemistry. This approach provided expression data for 8,728 transcripts and 102 proteins, differentially regulated in the infected human lymph node. Enhanced inflammation with upregulation of T-helper1-related genes, combined with marked dysregulation of matrix metalloproteinases, indicates tissue damage due to high immunoactivity at infected niche. This expression signature was accompanied by significant upregulation of an immunoregulatory gene, leukotriene A4 hydrolase, at both transcript and protein levels. Comparative transcriptional analyses revealed LNTB-specific perturbations. In contrast to pulmonary TB-associated increase in lipid metabolism, genes involved in fatty-acid metabolism were found to be downregulated in LNTB suggesting differential lipid metabolic signature. This study investigates the tissue molecular signature of LNTB patients for the first time and presents findings that indicate the possible mechanism of disease pathology through dysregulation of inflammatory and tissue-repair processes.

Extrapulmonary tuberculosis (EPTB) refers to infection of *Mycobacterium tuberculosis* at body sites other than the lungs^1^. Lymph node TB (LNTB) is one of the most common manifestations of EPTB that affects, most frequently, the peripheral and cervical lymph nodes. EPTB is reported to occur in isolation or along with the more frequent clinical presentation of TB, pulmonary TB (PTB)^2^,^3^. EPTB prevalence is reported from 15 to 20% of all cases of TB in endemic countries and accounts for more than 50% cases in immunocompromised individuals^3^,^4^,^5^. In 2012, 0.8 million out of total 6.1 million notified TB cases had EPTB^1^. While several genes and pathways are implicated in host response to PTB^6^, our understanding of EPTB manifestations is still very limited.

An important aspect of understanding host–pathogen interactions is to recognize the host tissue response to the infection and identify causal factors associated with disease manifestation. Although different studies have highlighted risk factors and cytokine expression for various forms of EPTB^7^,^8^,^9^,^10^, the knowledge of pathogen-related tissue expression changes in host is quite inadequate. So far, only few reports have studied the human tissue response in TB patients^11^,^12^. Therefore, in the present work, we aimed to examine the molecular response in infected tissue of LNTB patients to better comprehend the pathophysiological changes associated with EPTB. We employed global expression profiling by means of microarray and quantitative proteomics of infected lymph nodes and also performed comparative transcriptome analyses with publicly available tissue expression datasets of PTB-infected lung granulomas, tuberculous meningitis (TBM) brain tissue and lymph node cancer tissue to understand LNTB etiology.

## Results

### Patient characterization

Patients displaying cervical lymphadenopathy clinically suspected to be of tuberculous origin were prospectively recruited for this study at Rajan Babu Institute of Pulmonary Medicine and Tuberculosis (RBIPMT), Delhi, India. Patients of both sexes and all ages were included in the study (Table 1). The duration of symptoms at the time of first clinical presentation varied from 4 to 6 weeks and the primary complaints were swelling near the neck region, fever, night sweating, anorexia, and weight loss. Patients with human immunodeficiency virus infection, diabetes, previous history of any form of TB, and known malignancy were excluded from the study to avoid any bias in results.

**Figure 1:**
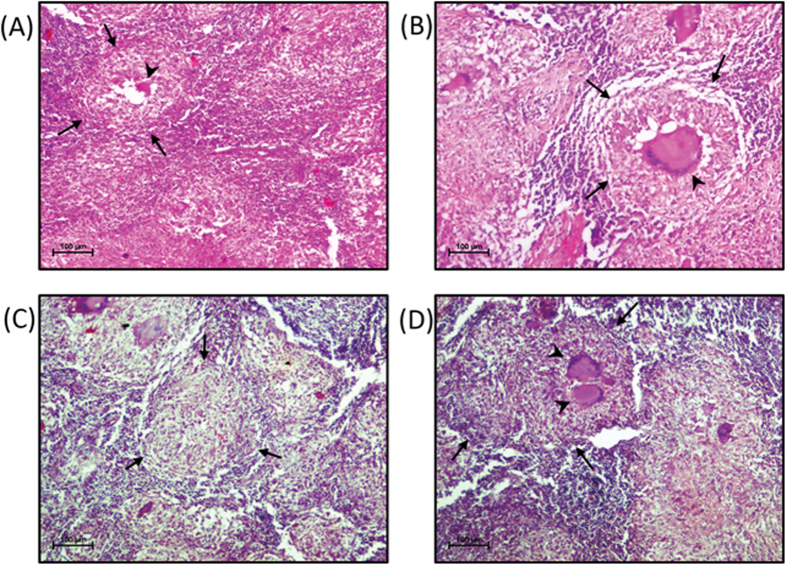
Hematoxylin & eosin staining for identification of granulomatous structures. Hematoxylin & eosin stained section of patient lymph node infected with *M*. *tuberculosis*, showing TB characteristic granuloma with Langhans giant cells at 10×. (**A**) Granuloma (marked by arrows) and Langhans giant cells (highlighted by arrowheads). (**B**) Granuloma (marked by arrows) surrounded by lymphoid cells with Langhans giant cell in the centre. (**C**) Granuloma (marked by arrows) surrounded by lymphoid cells. (**D**) Granuloma (marked by arrows) and Langhans giant cells (highlighted by arrowheads).

A total of 37 patients fulfilling the above mentioned criteria were enrolled over a period of two years for the study. Fine-needle aspirate was obtained from all the patients and subjected to smear microscopy for detection of *M. tuberculosis acid-fast* bacilli (AFB). Twenty-four patients had positive findings on AFB staining and were immediately put on anti-TB drugs; the remaining 13 were further subjected to excisional biopsy. The tissue fragments of these 13 patients were subjected to histopathological examination and tested positive for tuberculous granuloma (Fig. 1). Culturing of *M*. *tuberculosis* obtained from fluid of these 13 patients was also employed for confirmation. These patients constituted the case group for this study and consisted of twelve females and one male with a mean age of 16 (9–26) years (Table 1).

### Transcriptional profiling of human lymph node tissues

**Figure 2:**
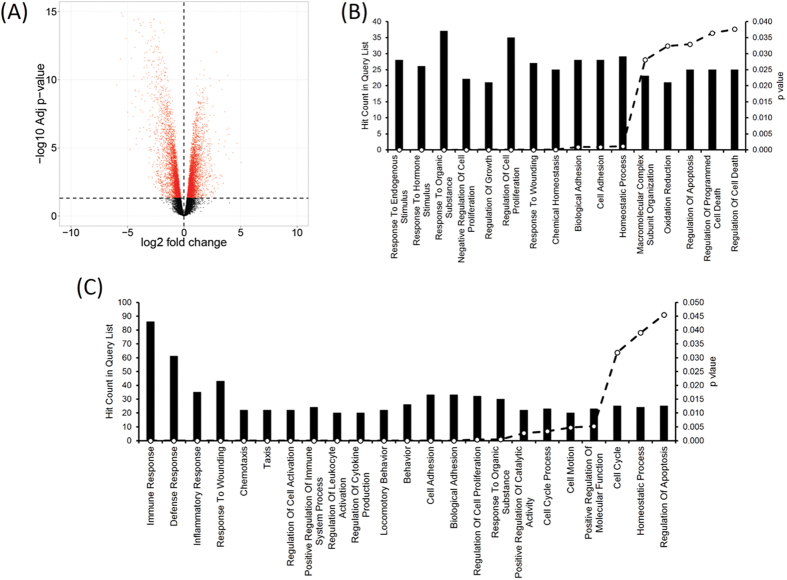
Transcriptomic profiling of LNTB infected tissue. (**A**) Volcano plot showing differentially expressed genes (DEGs) in microarray analysis of LNTB infected tissue with respect to control tissue. The negative log_10_ transformed false discovery rate (FDR) adjusted *p-*values are plotted against the average log_2_ fold changes in expression (*x* axis). DEGs were determined using *limma* followed by FDR correction. Horizontal dashed line indicates the threshold for significance (FDR adjusted P < 0.05) and vertical dashed line indicates the upregulated (right side) and downregulated (left side) probes. (**B**,**C**) Functional enrichment analyses of top 10 percent downregulated (**B**) and upregulated (**C**) DEGs in DAVID. The bar graph shows the number of DEGs observed in each gene ontology (GO) category (atleast 20 genes per category), corresponding p-values are represented in the secondary axis.

We carried out comparative transcriptomic analyses of thirteen patient and two healthy lymph nodes using whole human genome oligonucleotide arrays. From this analysis, we identified 10,125 probes to be significantly differentially expressed (adjusted p-val < 0.05, fold value ± 1.2 ( ≈ logFC ± 0.27), of which 5,497 probes accounting for 4,875 genes had lower expression values than control and 3,949 genes represented by 4,628 probes showed expression values higher than the control group (Fig. 2A, Supplementary Table ST1, GEO accession: GSE63548). The relationship between gene expression profiles was examined by principal component analysis of normalized gene expression data of samples and unsupervised hierarchical clustering using the top 50 genes most variant across the dataset (Supplementary Figure S1,S2). In order to functionally characterize the differentially expressed transcripts, GO based classification was carried out using the DAVID bioinformatics resources^13^, for the downregulated and upregulated gene set (Fig. 2B,C). The most downregulated pathways were the response to endogenous stimulus, regulation of cell proliferation and chemical homeostasis (Fig. 2B). The most upregulated biological processes included immune response, inflammatory response, and chemotaxis (Fig. 2C). Supplementary Table ST2 lists the GO biological process annotations enriched at FDR 0.05 for the expression data.

### Host transcriptional response at active site of infection

We performed MetaCore^™^ analysis of differentially expressed genes and chose the most upregulated biological process obtained through DAVID, the immune response pathway. The key pathways studied through this analysis were namely Th1 and Th2 cell differentiation, Interferon-gamma (IFN-γ) signaling and TNF-α signaling. An increase in *NFKB* expression (2.5-fold) (Supplementary Table ST1), a key regulator of the innate immune response^14^, was observed along with several genes (*STAT1*, *IFNG*, *TNF*, *CXCR3* and *STAT4*; 15.4-, 5.8-, 4.4-, 2.8- and 2.4-fold, respectively) in Th1 subset. Interestingly, this increase in Th1 profile was accompanied by downregulation of *c-MAF* (−2.5-fold), a key transcription factor of Th2 pathway, known to upregulate *IL-10* gene expression^15^ and *FOXP3*, regulator of regulatory T (T_reg_) cells (Supplementary Figures S3–S5). The enhanced inflammatory response was also highlighted by downregulation of *c-MYC* (−2.1-fold), known for its regulatory role in cell proliferation and inflammation^16^, and peroxisome proliferator-activated receptor gamma (PPARγ, 7.9-fold) in LNTB patients (Supplementary Table ST1). PPARγ signaling, known to regulate glucose metabolism and anti-inflammatory effects^17^, is used by mycobacteria to circumvent host response to facilitate its survival^18^. In view of the anti-inflammatory property and downregulation at site of infection in LNTB, further characterization of PPARγ in EPTB is warranted.

Using the whole genome transcriptional profile, we gained an understanding of factors that play an active role during infection. Our analysis revealed a strong IFN-inducible signature (*OAS1*, *IFI6*, *IFI44*, *IFI44L*, *OAS3*, *IRF7*, *IFIH1*, *IFI16*, *IFIT3*, *IFIT2*, *OAS2*, *GBP1*, *GBP5*, *GBP2*, *TAP1*, *STAT1*, *STAT2*, *IFI35*, *TAP2*, *CD274*, *CXCL10*, *IFIT5*) in infected tissue (Supplementary Table ST1). Interestingly, on comparing the 393-transcript signature obtained by analysis of PTB patients’ blood in a previous study^19^, we observed a core set of 171 genes to be upregulated in our analysis (Supplementary Table ST3), indicating involvement of some common host effector functions in response to mycobacterial infection.

Another interesting feature was the differential expression of matrix metalloproteinases (MMPs)/tissue inhibitors of MMPs (TIMPs) in LNTB patients. MMPs/TIMPs are associated with tissue damage/inflammation as well as matrix remodeling^20^. While increased expression of MMP9 (3.6-fold) and MMP12 (10-fold) has been reported earlier in TB patients^7^, expression of TIMP2, TIMP3, TIMP4 were also found significantly reduced in this study (−2.27-, −8.46-, −69.7-fold, respectively), highlighting the dysregulation in remodeling of extracellular matrix during LNTB. Another example of differentially regulated genes at infected site included elevated expression of pro-apoptotic caspases (*CASP3, CASP7, CASP10*), inflammatory caspases (*CASP1, CASP4, CASP5*) and pro-necrotic genes (*RIPK3*, *MLKL*) (Supplementary Table ST1). The host cell death response critically influences the fate of bacterial infection. Thus, operation of diverse cell death mechanisms through these genes might be a survival strategy employed by the bacterium.

### Protein profiling of human lymph node tissues and correlation analysis

**Figure 3:**
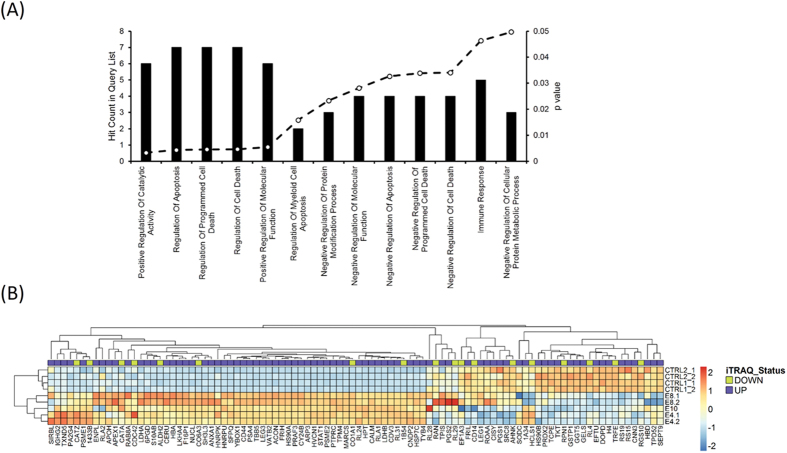
Proteomic profiling of LNTB infected tissue. (**A**) Functional enrichment analyses of differentially expressed proteins generated using DAVID. The bar graph displays the number of overexpressed proteins observed in each GO category. All the categories presented in the graph have P-values < 0.05. (**B**) Heat map showing gene expression status of significantly variant proteins in proteomic profiling of LNTB tissue. Blue = low expression, red = high expression.

We also carried out protein profiling of one healthy lymph node and three LNTB-infected tissues and implemented a relative quantitative proteomics approach using isobaric tag for relative and absolute quantitation (iTRAQ)^21^,^22^ and detected a total of 459 proteins in the samples (Supplementary Table ST4). However, on consideration of significance and consistency across all three samples, 82 proteins were found to be more abundant in the infected lymph node tissue, while 20 proteins were less abundant as compared to control tissue (Supplementary Table ST5). The GO based classification using DAVID revealed proteins belonging to biological processes like regulation of catalytic activity, apoptosis and immune response to be significantly upregulated (Fig. 3A).

To ascertain the relationship between the transcript expression and protein abundance, we compared the differential expression ratios observed in the two datasets. The correlation obtained for the genes for which both transcript and protein levels were measured was, however, not significant (Spearman correlation 0.09, p-value 0.4) (Supplementary Figure S6). Discordant expression of corresponding protein and mRNA has been observed previously in disease pathologies such as lung adenocarcinomas^23^. To detect the global structure of the data, the expression of genes measured at both the transcript and protein levels was subjected to hierarchical clustering (Fig. 3B). Upon integration of both data, we found that out of 102 differentially regulated proteins, 46 were co-regulated at the transcript level. This combined approach revealed three interesting candidates related to inflammatory response. These were leukotriene A4 hydrolase (LTA4H/LKHA4, 2.1-fold), V-type proton ATPase subunit B (ATP6V1B2/ VATB2, 2.1-fold) and proteasome activator complex subunit 2 (PSME2, 4.1-fold) (Supplementary Tables ST1, ST5).

### Comparative transcriptome meta-analysis

**Figure 4:**
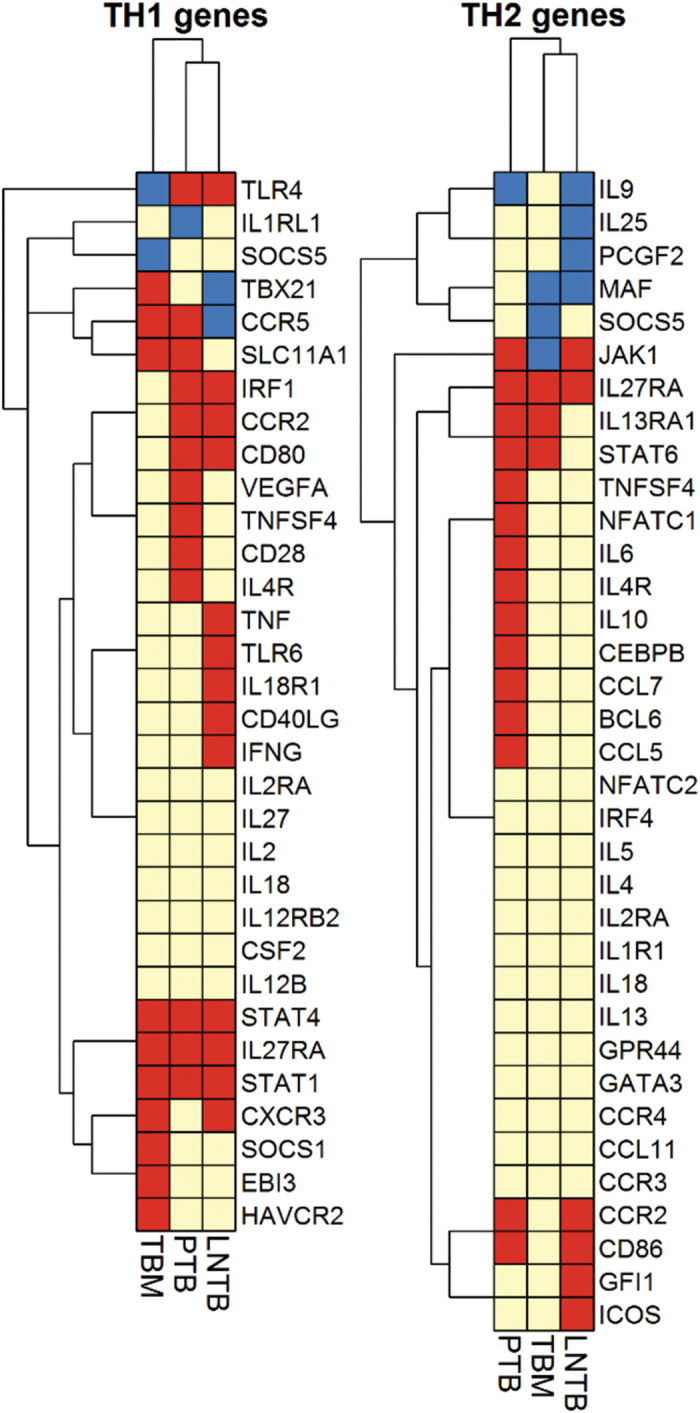
Qualitative heat map analysis of Th1/Th2 pathway genes in LNTB, PTB and TBM. Heat map of the expression of genes belonging to the Th1 and Th2 subset, (subset mentioned in Qiagen SABiosciences online resource). Microarray datasets of current study (LNTB), PTB (GSE20050) and TBM (GSE23074) were considered for this analysis. The results presented represent the differential gene expression status in the disease in relation to the controls chosen in respective studies. Unsupervised hierarchical clustering was performed. Upregulated genes are marked by the red boxes; downregulated genes by blue boxes and yellow boxes indicate lack of differential expression.

An *in silico* transcript analysis using GEO microarray expression datasets of PTB lung granuloma tissue, TBM brain tissue and lymph node cancer (LNC) tissue was carried out in order to have a comparative disease-associated host expression analysis. The LNC dataset was included to analyze the major biological processes affected in these two different pathologies of the same region. While processes related to cell cycle control and proliferation were significantly affected in case of LNC, the most significant process affected in LNTB was immune response (Supplementary Table ST2). Comparative gene expression analysis revealed that inflammatory response to LNC and LNTB were different, reiterating the fact that even if same tissue is host for different diseases, immunopathology is unique (Supplementary Figure S7). We also examined the Th1/Th2 compartmentalization in LNTB, TBM and PTB by comparison of expression datasets. While PTB displayed a mixed Th1/Th2 response, we observed a strong and exclusive association of Th1 effector function with LNTB and TBM (Fig. 4).

We also analyzed the status of fatty acid metabolism genes in these datasets since lipid metabolism has been significantly associated with *M. tuberculosis* pathogenesis. We found that unlike the PTB lung tissue^11^, key genes involved in host lipid synthesis, namely adipophilin/ perilipin 2 (*PLIN2,* −2.1-fold) and *ACSL1* were downregulated with respect to normal tissue (Supplementary Table ST1). We did observe upregulation of some genes such as encoding AMP-activated protein kinase, which is activated in inflammatory conditions to regulate catabolic processes^24^, and *ACOT7* signifying their role in energy homeostasis (Supplementary Figure S8). We carried out MetaCore^TM^ analysis for fatty acid metabolism pathway and noted that most of the genes are not differentially expressed, apart from the stark downregulation of fatty acid synthase (−15.6-fold), a key nodal component in fatty acid synthesis (Supplementary Figure S8). These results suggest regulation of specific fatty acid metabolism genes required for basal energy maintenance at the site of infection in LNTB.

### Validation of selected targets by RT-PCR and Immunohistochemistry

**Figure 5:**
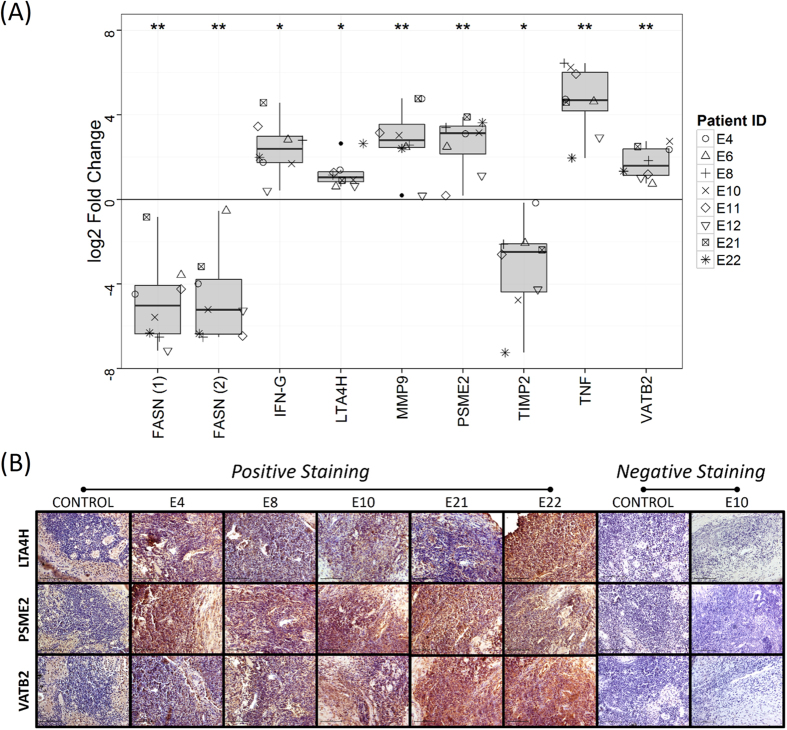
Validation of selected targets by Quantitative real time PCR and Immunohistochemistry. (**A**) Quantitative real time PCR of VATB2, FASN, IFN, LTA4H, MMP9, PSME2, TIMP2 and TNF mRNA levels. All fold changes were derived using the formula, 2^−(ΔΔCP)^, and patient IDs are indicated in adjoining box. Statistical significance of log2 fold change as determined using one sample t-test is shown on the top of each boxplot by “*” for p-value < 0.05, “**” for p-value < 0.001. (**B**) Immunohistochemical analysis of lymph node sections from LNTB patients and healthy controls. Staining was performed with anti-LTA4H, anti-PSME2 and anti-VATB2 antibodies followed by colorimetric detection. Positive staining (brown; diaminobenzidine staining) and negative staining (blue; nuclear hematoxylin staining) are shown. Negative control was included without primary antibody incubation. Magnification: 40×

The candidates chosen by gene-protein integrative analysis, LTA4H, ATP6V1B2 and PSME2, were subjected to further validation. Few other targets were also selected based on their role in inflammation, matrix degradation and fatty acid metabolism (*IFNG*, *TNF, FASN, MMP9* and *TIMP2)* and validated by performing qRT-PCR (Fig. 5A). One control sample and eight patient samples (three independent patient samples not used in microarray experiment) were chosen. The trend observed in cDNA microarray was confirmed in all genes, with statistical significance of log2 fold change difference (P < 0.05 by one sample t-test). Upregulation of proteins LTA4H, ATP6V1B2 and PSME2 was also confirmed by immunohistochemical analysis in five patient samples, including two independent ones, which were not used in the iTRAQ study (Fig. 5B). All the three proteins showed uniform abundance in the entire lymph node section.

## Discussion

One-third of the world’s population is infected with *M*. *tuberculosis*, and in 10%–15% of cases, reactivation occurs at extrapulmonary sites without active PTB^25^. An impediment in study of EPTB is accurate patient diagnosis, as the associated clinical symptoms overlap with other pathologies^3^ and no precise diagnostic or markers are currently available. *M*. *tuberculosis*–specific physiological changes in humans can be better understood by exploring the host tissue response induced at the disease site^26^. Lymph nodes are the first conduits of immune cell migration following TB infection and are the most common site of infection in EPTB^27^,^28^. Therefore, we sought to reveal physiological changes that accompany LNTB progression through global expression profiling of lymph nodes from LNTB patients.

T-cell activation is central to the protective immunity to *M*. *tuberculosis* infection, and cytokines play an important role in driving T cell differentiation. The interplay between pro-inflammatory cytokines (Th1), anti-inflammatory cytokines (Th2), and chemokines is significant in governing the outcome of disease. Past evidence suggest ambiguous role of Th2 response in protection against TB^29^, but a recent report supports the view that both Th1 and Th2 responses are induced in an optimal anti-TB-response^30^. Our expression profile indicated a proinflammatory response with marked activation of IFN signaling. We also observed downregulation of transcription factors, *c-MAF* and *FOXP3*, key regulators in function of Th2 and T_reg_ cells, respectively^15^,^31^. Foxp3^+^ T_reg_ cells are important for controlling excessive inflammation, but can also hamper bacterial clearance by immunosuppressive function^31^. Role of Th2 and T_reg_ effector cells in EPTB infection is still not well established, and in view of present findings, should be explored further.

Genes involved in extracellular matrix remodeling play a critical role in infection process^20^. Mycobacteria-triggered expression of MMP9 has been seen to enhance nascent granuloma maturation and bacterial growth^32^. Upregulation of MMP-9 and MMP-12 in LNTB patients further suggests an active role of these enzymes in TB infection process. On the other hand, downregulation of TIMPs might be a pathogen-induced event and further characterization of the MMP/TIMP balance in tissue pathology may be beneficial for therapeutic strategies. Host cell death is an intrinsic immune defense mechanism in response to microbial infection. Differential regulation of genes belonging to diverse pathways such as apoptosis, pyroptosis and necroptosis suggest pathogen-driven manipulation of cell death at infected site. Future studies on the cell death response during host–pathogen interactions will provide better understanding of these mechanisms.

Three molecules (LTA4H, PSME2 and ATP6V1B2) chosen for validation from the gene-protein integrative analysis are known to play key roles during inflammatory response^33^,^34^,^35^. LTA4H function as an immunoregulatory switch and variation in the level of LTA4H is known to influence the balance between proinflammatory leukotriene B4 and anti-inflammatory lipoxin A4^33^. It is known that an excess of LTA4H leads to a hyperinflammatory state and upregulation of this molecule in LNTB patients is reflective of such a situation. In view of earlier studies and our results, LTA4H may play a role in TB susceptibility. PSME2 is implicated in the immunoproteasome assembly and is upregulated in response to inflammatory conditions persistent in diseases such as tularemia, a bacterial disease related in pathology to TB^34^. Upregulation of PSME2 in LNTB tissue, thus, implicates a similar role of gene in diseases with related etiologies. ATP6V1B2 encodes a component of vacuolar ATPase (V-ATPase). Upregulation of ATP6V1B2 has been implicated in apoptosis inhibition in pigmented villonodular synovitis, a joint disease with tumorous and inflammatory features^35^. Interestingly, genes encoding seven different subunits of V-ATPase were also upregulated in LNTB.

Kim *et al.* had shown *M. tuberculosis*-mediated upregulation of host lipid metabolism genes in infected lung tissue granulomas^11^. However, unlike PTB, we did not find any realignment in the expression of majority of fatty acid metabolism genes in LNTB. Moreover, our analysis revealed downregulation of key central regulators, suggesting a different tissue-specific signature. It is, however, important to note that the earlier study involved enrichment of granuloma tissue region. Nonetheless, downregulation of metabolism-associated genes indicates tissue response to lipid accumulation varies with the site of infection.

A comprehensive network modeling of host response to TB suggested possible biomarkers for disease, based on high activity of network nodes in the infection process^36^. Many of these candidate genes were also upregulated in the present study, namely, *VAMP5*, *CASP5*, *OAS1*, *IFL30*, *IRF7*, *GNG10*, *PYCARD*, *IRF9*, *HLA*-F and *NCF*-*1*. This network of genes may be studied further to probe their potential as biomarkers.

One of the limitations of our study was the modest cohort size. We tried to circumvent this problem by validating expression levels of candidate genes in independent samples by RT-PCR and IHC to prevent any data bias. Moreover, this high-throughput experimental approach can be complemented with associated long-term clinical annotation comprising drug response and expression changes in patients. So, this work for the first time provides a snapshot of expression profile of infected human lymph node tissues, both at transcript and protein level obtained from the same sample. It expands the knowledge base on the relative contribution of host-related factors to the pathogenesis of TB. It is known that host’s local tissue response to infection drives the disease progression and a low Th2/Th1 ratio may result in increased inflammation and thus, susceptibility to TB. Our data argues against the presumption that EPTB is a marker of underlying immunodeficiency^37^. The results support the critical role played by the Th1 subset in host response to TB. Our results show that the inflammatory conditions and dysregulation of cell repair mechanisms are factors related to development of both LNTB and PTB, while factors such as fatty acid metabolism might have a more tissue-related relevance. It will be interesting to look for tissue specific signature in other TB manifestations, which if corroborative will have substantial clinical impact.

## Methods

### Human Tissues

The study was carried out as per protocols approved by institutional ethics committee at CSIR-Institute of Genomics and Integrative Biology, Delhi and RBIPMT, Delhi, India. All the methods were carried out in accordance with the approved guidelines. Informed written consents were obtained from all the patients. Human lymph node tissue samples were surgically excised from patients displaying swelling in the cervical lymph node region. The aspirate obtained during biopsy was sent to diagnostic division for culture testing of *M*. *tuberculosis.* Each biopsy lymph node sample was cleared in phosphate-buffered saline and a part was kept in pathology division for histopathological examination and tested for presence of other complications (e.g. malignancy). The excised lymph node samples were further divided into three parts for research purpose (i) one portion was fixed with 10% formalin and embedded in paraffin for immunohistological examination (ii) second portion collected in RNAlater^®^ solution was preserved at −80 °C for use in transcriptomics study and (iii) the third portion was snap-frozen for the proteomics study. The transport time for samples to reach BSL3 research laboratory was less than thirty minutes. The paraffin embedded samples were stained with hematoxylin & eosin (H&E) and Ziehl-Neelsen stain as per standard protocols. For control tissue, two healthy lymph node RNA samples were purchased (Life Technologies, CA, USA) for the transcriptomics study, and two healthy lymph node protein lysate samples were purchased (Abcam, MA, USA) for proteomics study. Also, four sets of lymph node tissue sectioned samples were purchased (Abcam, MA, USA) for immunohistochemistry analyses.

### Transcriptional Profiling

Total RNA was extracted from all the lymph node tissue samples using the manual TRIzol^®^ (Invitrogen) method in BSL3 facility as per protocol described previously^38^. Labeled cRNA was hybridized onto Illumina HumanHT−12 v4 Expression BeadChip arrays containing more than 47,000 probes representing more than 31,000 annotated genes. Hybridization and washing steps were performed as per manufacturer’s instructions. The arrays were scanned by Illumina iScan System. We generated global gene expression profile for two controls and thirteen tissue biopsy samples.

### Microarray Data Analysis

Background subtracted expression values for probes (Sample Probe Profile) were exported from Illumina Gene expression version 1.9.0 and supervised normalization of microarrays was used to generate normalized expression values for the samples^39^. *limma* was then employed to detect differential expression among probes, and false discovery rate (FDR) adjusted p-values were considered for downstream analysis.

For comparative meta-analysis of global transcriptome profiles, SOFT (Simple Omnibus Format in Text) data files for GSE20050^11^, GSE23074^40^ and GSE51985^41^ were downloaded from the NCBI Gene Expression Omnibus (GEO) server. Unless normalized data was directly available in the GEO SOFT files, normalization based on quantiles was performed on all datasets using the *preprocessCore* package in R Bioconductor. Differentially expressed genes for these data were evaluated using *limma* in accordance with the sample information as provided in the respective GEO project page. The various gene panels used in this study were obtained from SABiosciences online resource (http://www.sabiosciences.com/ArrayList.php). The functional annotation tool, Database for Annotation, Visualization and Integrated Discovery (DAVID) and MetaCore^TM^ pathway analysis server were used for Gene Ontology (GO) based functional enrichment and pathway annotations.

### Protein profiling using iTRAQ

Total protein lysates were extracted from lymph node tissue samples (100mg) of three patients. The iTRAQ experiment was customized through Proteomics International (PI) Perth, Australia. Spectral data was analyzed against the Swiss-Prot database using Protein Pilot v4 Software (Life Technologies, CA, USA). Protein identifications were considered at global FDR of <0.1% and confidence level >95% with two or more peptide matches. For differential expression, average iTRAQ ratio for the 3 patients’ value relative to the control value was considered. A threshold cutoff of >1.2 fold change for upregulated proteins and <0.8 fold change for down regulated proteins was applied. Protein iTRAQ ratios with p-value <0.05, were considered significant.

### Quantitative real-time RT-PCR

One control and eight patient samples (including three independent patient samples not used in -omics experiments) were used for quantitative real-time RT-PCR (qRT-PCR) analysis. 50 ng of RNA was reverse transcribed into cDNA using Invitrogen Superscript III kit following manufacturer’s recommendations. RT-PCR was performed with LightCycler 480 System II 96 well plate (Roche Diagnostics) according to manufacturer’s protocols. The β-actin/18S rRNA genes were used for normalization and fold changes were calculated using the formula, 2^−(ΔΔCP)^^42^.

### Immunohistochemistry of human LNTB tissues

Paraffin embedded sections were processed for immunohistochemistry analysis as per standard protocol^12^, and treated with antibodies, anti-LTA4H, anti-PSME2 and anti-ATP6V1B2. The reaction was visualized with DAB-substrate kit (Vector laboratories) as per manufacturer’s instructions. The sections were counterstained with hematoxylin. Images were captured by Nikon Eclipse 90i automated research microscope System with NIS-Elements imaging software.

## Additional Information

**How to cite this article**: Maji, A. *et al.* Expression profiling of lymph nodes in tuberculosis patients reveal inflammatory milieu at site of infection. *Sci. Rep.* **5**, 15214; doi: 10.1038/srep15214 (2015).

## Supplementary Material

Supplementary Information

## Figures and Tables

**Table 1:** Details of LNTB patients enrolled in the study.

Patient ID	Age	Sex	H/E Staining	Culture	Current medication	History of TB	Microarray	iTRAQ
E-4	13	F	+ve	+ve	NO	NO	YES	YES
E-6	12	M	+ve	+ve	NO	NO	YES	
E-7	17	F	+ve	+ve	NO	NO	YES	
E-8	26	F	+ve	+ve	NO	NO	YES	YES
E-10	16	F	+ve	+ve	NO	NO	YES	YES
E-11	18	F	+ve	+ve	NO	NO	YES	
E-13	26	F	+ve	+ve	NO	NO	YES	
E-14	9	F	+ve	+ve	NO	NO	YES	
E-15	25	F	+ve	+ve	NO	NO	YES	
E-16	13	F	+ve	+ve	NO	NO	YES	
E-17	16	F	+ve	+ve	NO	NO	YES	
E-18	11	F	+ve	+ve	NO	NO	YES	
E-19	15	F	+ve	+ve	NO	NO	YES	
