# Supplementary material for: Expression profiling of lymph nodes in tuberculosis patients reveal inflammatory milieu at site of infection
Source: Sci Rep. 2015 Oct 15;5:15214. doi: 10.1038/srep15214 (PMC4606593; doi:10.1038/srep15214)
Supplement: Supplementary Information [file srep15214-s1.pdf]

Expression profiling of lymph nodes in tuberculosis patients reveal inflammatory milieu at site of infection

Abhijit Maji[1†], Richa Misra[1†‡], Anupam Kumar Mondal[1,2†], Dhirendra Kumar[1], Divya Bajaj[3], Anshika Singhal[1], Gunjan Arora[1,§], Asani Bhaduri[1,‖], Andaleeb Sajid[1,§], Sugandha Bhatia[1], Sompal Singh[4], Harshvardhan Singh[4], Vivek Rao[1], Debasis Dash[1], Baby Shalini E[5], Joy Sarojini Michael[5], Anil Chaudhary[6], Rajesh S. Gokhale[1], Yogendra Singh[1*]

Supplementary Information

Methods

Transcriptional Profiling

The quantity and quality of the isolated RNA was determined using Nanodrop-1000 (Thermo Fischer Scientific) and Agilent 2100 Bioanalyzer (Agilent Technologies, CA, USA), respectively. RNA with RIN (RNA integrity number) value >7 was considered for further assessment as it reflects ideal value of integrated RNAs. These RNA samples had both 260/280 nm absorbance and 28S/18S rRNAs peak ratio greater than two. To obtain sufficient quantities of RNA for microarray, two-rounds of linear amplification of RNA were conducted using illumina TotalPrepTM RNA Amplification Kit (Applied Biosystems by Life Technologies, CA, USA). Biotinylated, purified antisense RNA was quantitated and 750ng of labeled cRNA was hybridized onto Illumina HumanHT-12 v4 Expression BeadChip arrays.

Protein profiling using iTRAQ

Lymph node tissue samples of three patients (100mg) were lysed in 10 volumes of buffer containing Urea (6M), thiourea (2M), 2% CHAPS, 0.5% SDS, homogenized and centrifuged at 13,000 rpm for 10 min at 4°C. The extracted supernatant was filtered to remove any infectious agent. Sonication was performed for 10 min to shear all DNA, followed by

centrifugation at 13000 rpm for 10 at 4°C. Supernatant was collected and precipitated with ice-cold acetone. The final pellet was resuspended in 6M urea and 50mM triethyl ammonium bicarbonate (TEAB) and protein quantitation was carried out by DC protein assay (Biorad, CA, USA). The iTRAQ experiment was customized through Proteomics International (PI) Perth, Australia.

Immunohistochemistry of human LNTB tissues

Paraffin embedded sections were heat-fixed, deparaffinized and rehydrated. Endogenous peroxidase activity was quenched by 3% H_2O_2 for 30 min at room temperature. Epitopes were heat-retrieved in 1mM EDTA buffer pH 8.0 for 15 min, and tissue sections were blocked with 1% BSA in 0.1% PBST at room temperature for 2h. The sections were subjected for overnight incubation at 4°C with monoclonal primary antibodies at following dilutions- anti-LTA4H (dilution 1: 250, Abcam ab133512), anti- PSME2 (dilution 1: 150, Abcam ab58123), anti- ATP6V1B2 (dilution 6.5ug/ml Abcam, ab129320). After three washes with PBST, poly HRP-conjugated secondary antibodies (goat anti-rabbit IgG, HPO20; rabbit anti-mouse IgG, 105501, Bangalore Genei) were incubated for 1h. The reaction was visualized with DAB-substrate kit (Vector laboratories) as per manufacturer's instructions. The sections were counterstained with hematoxylin. Sections without primary antibody were included as negative control. Images were captured by Nikon Eclipse 90i automated research microscope System with NIS-Elements imaging software.

Sequence for the RT primer sets are as follows:

ATP6V1B2 F.P	GCACTTTGCAGTGGAAAACA
ATP6V1B2 R.P	GACGGAGTAGGCAATGGAAA
PSME2 F.P	CCCCAAGATTGAAGATGGAA
PSME2 R.P	TCCTCCTCCTGGTTCATGTC
LTA4H F.P	TTCTTCTCAGCTGTGTCCGT
LTA4H R.P	ACCAAACAATCGTCCGCAAA
TIMP2 F.P	AAGCGGTCAGTGAGAAGGAAG
TIMP2 R.P	GGGGCCGTGTAGATAAACTCTAT
MMP9 F.P	TGTACCGCTATGGTTACACTCG

MMP9 R.P	GGCAGGGACAGTTGCTTCT
FASN F.P	AAGGACCTGTCTAGGTTTGATGC
FASN R.P	TGGCTTCATAGGTGACTTCCA
FASN2 F.P	CACAGGGACAACCTGGAGTT
FASN2 R.P	ACTCCACAGGTGGGAACAAG
STAT1 F.P	CGGCTGAATTTCGGCACCT
STAT1 R.P	CAGTAACGATGAGAGGACCCT
IFNG F.P	TCGGTAACTGACTTGAATGTCCA
IFNG R.P	TCGCTTCCCTGTTTTAGCTGC
TNF F.P	CCTCTCTCTAATCAGCCCTCTG
TNF R.P	GAGGACCTGGGAGTAGATGAG
STAT4 F.P	GCTTAACAGCCTCGATTTCAAGA
STAT4 R.P	GAGCATGGTGTTCATTAACAGGT
Beta-actin F.P	CGTCTTCCCCTCCATCG
Beta-actin R.P	CTCGTTAATGTCACGCAC
18S F.P	GTAACCCGTTGAACCCCATT
18S R.P	CCATCCAATCGGTAGTAGCG

Supplementary Figure S1

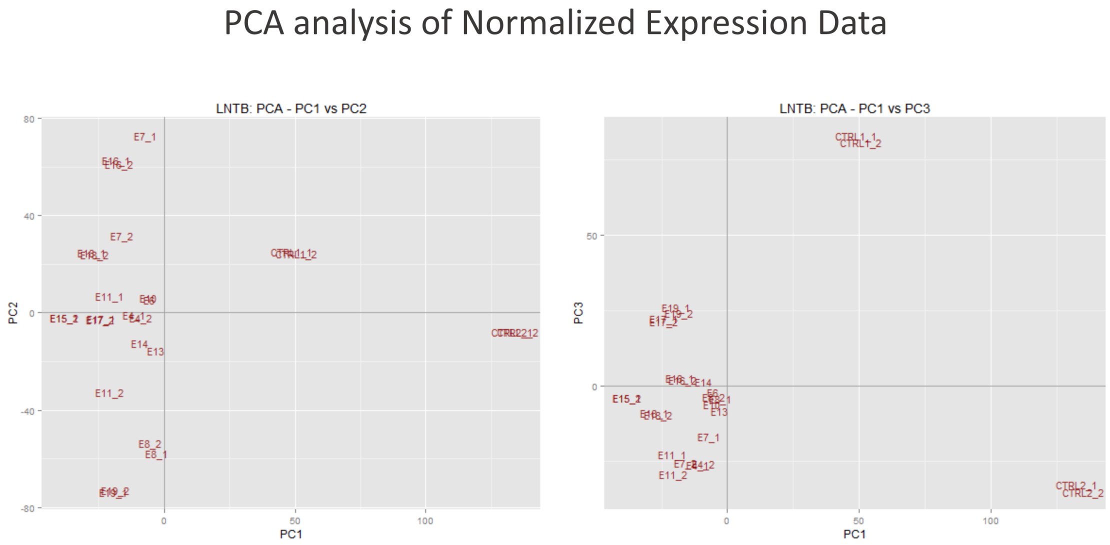

Supplementary Figure S1: **Principal component analysis (PCA) of normalized gene expression data of LNTB patient and control samples**.

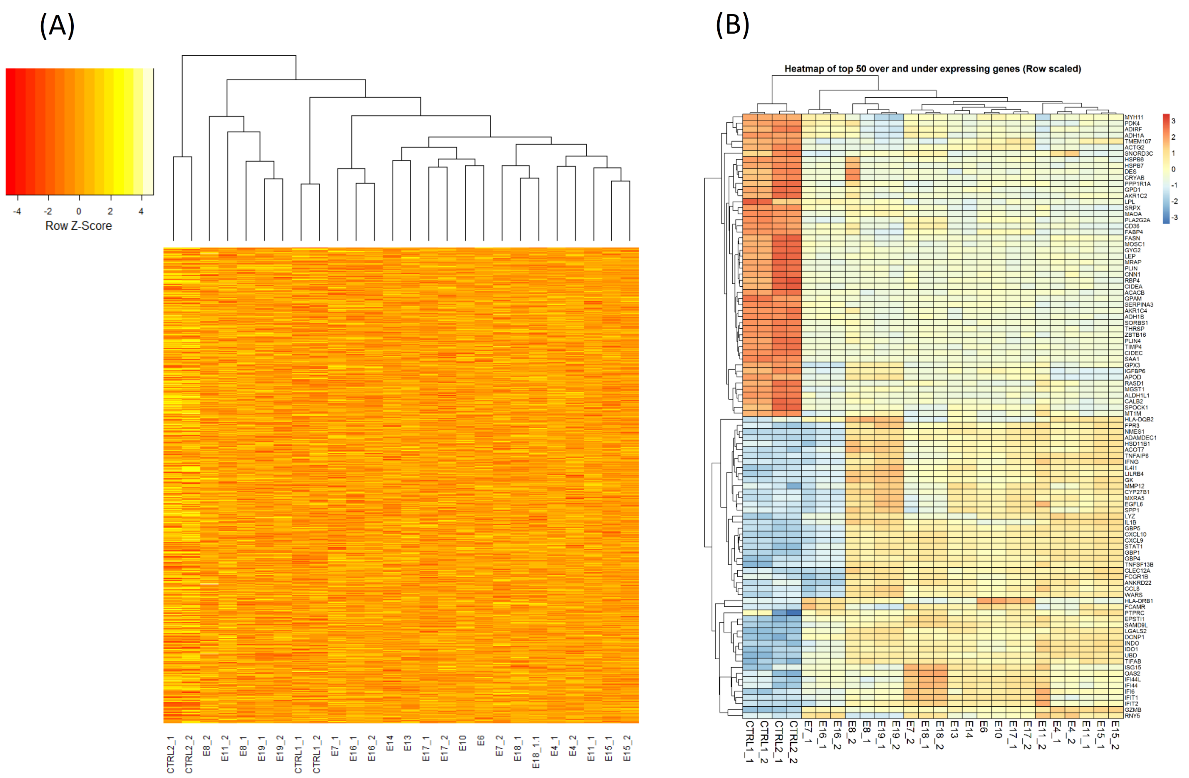

Supplementary Figure S2: ***Heat map of differentially expressed genes in LNTB*. (A)** Heat map depicting the normalized expression values of all probes (rows) across all samples (columns). The legend on the left depicts row scaled color-coded expression values. Dendrogram depicts unsupervised hierarchical clustering of patient and control samples. **(B)** Heat map representation of the normalized gene expression of top 50 differentially expressed genes (upregulated and downregulated) between LNTB patients and healthy individuals. Each column represents a sample, technical replicates are marked by numbers 1 and 2 following their patient ID. Gene symbols are indicated at the right of the heat map.

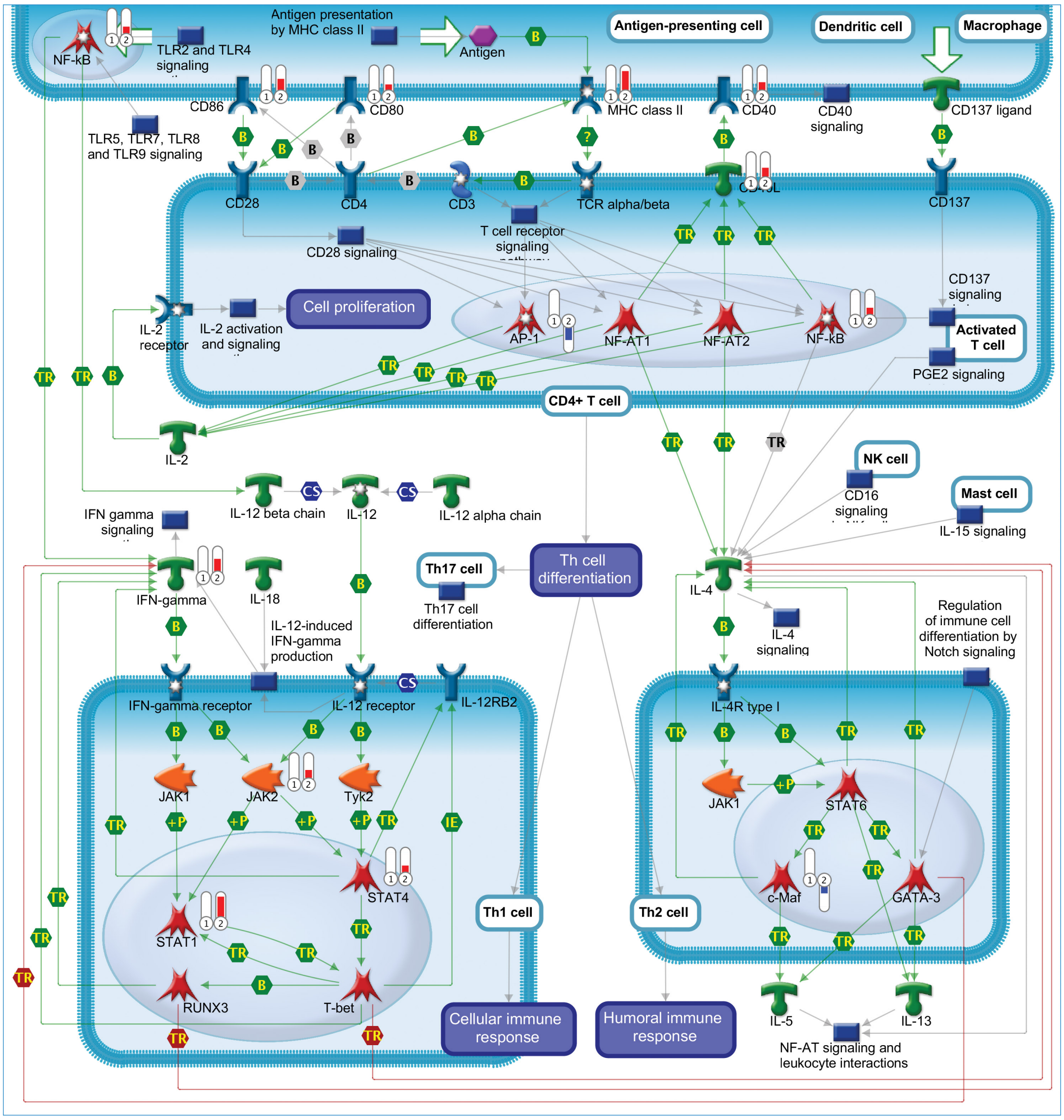

Supplementary Figure S3: ***MetaCore^{TM} analysis of process Immune response-Th1 and Th2 cell differentiation.*** Differentially expressed genes are shown with two bars (1 and 2) by their side, where the first bar represents the status of the control set and the second depicts the cumulative status of LNTB patients. If a gene exhibits overexpression in patients as compared to control, the second bar (bar 2) contains red fill, and the height of fill denotes extent of overexpression, on the contrary, genes with less expression in patients than controls, contain blue color in their second bar (bar 2). Detailed legend for symbols used in this image can be found at www.pathwaymaps.com/pdf/MC_legend.pdf.

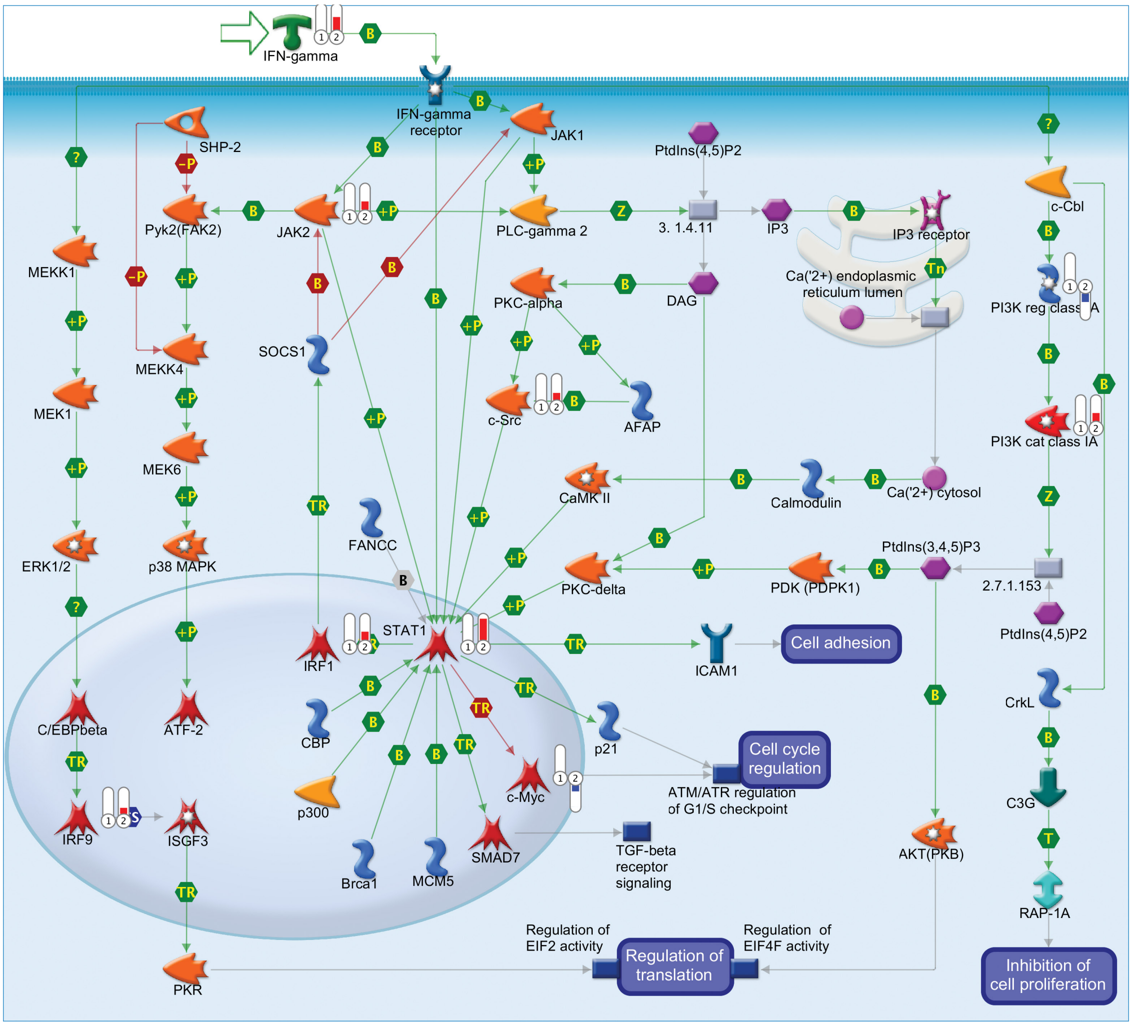

Supplementary Figure S4: ***MetaCore^{TM} analysis of process Immune response- IFNγ signalling pathway.*** Differentially expressed genes are shown with two bars (1 and 2) by their side, where the first bar represents the status of the control set and the second depicts the cumulative status of LNTB patients. If a gene exhibits overexpression in patients as compared to control, the second bar (bar 2) contains red fill, and the height of fill denotes extent of overexpression, on the contrary, genes with low expression in patients than controls, contain blue colour in their second bar (bar 2). Detailed legend for symbols used in this image can be found at www.pathwaymaps.com/pdf/MC_legend.pdf.

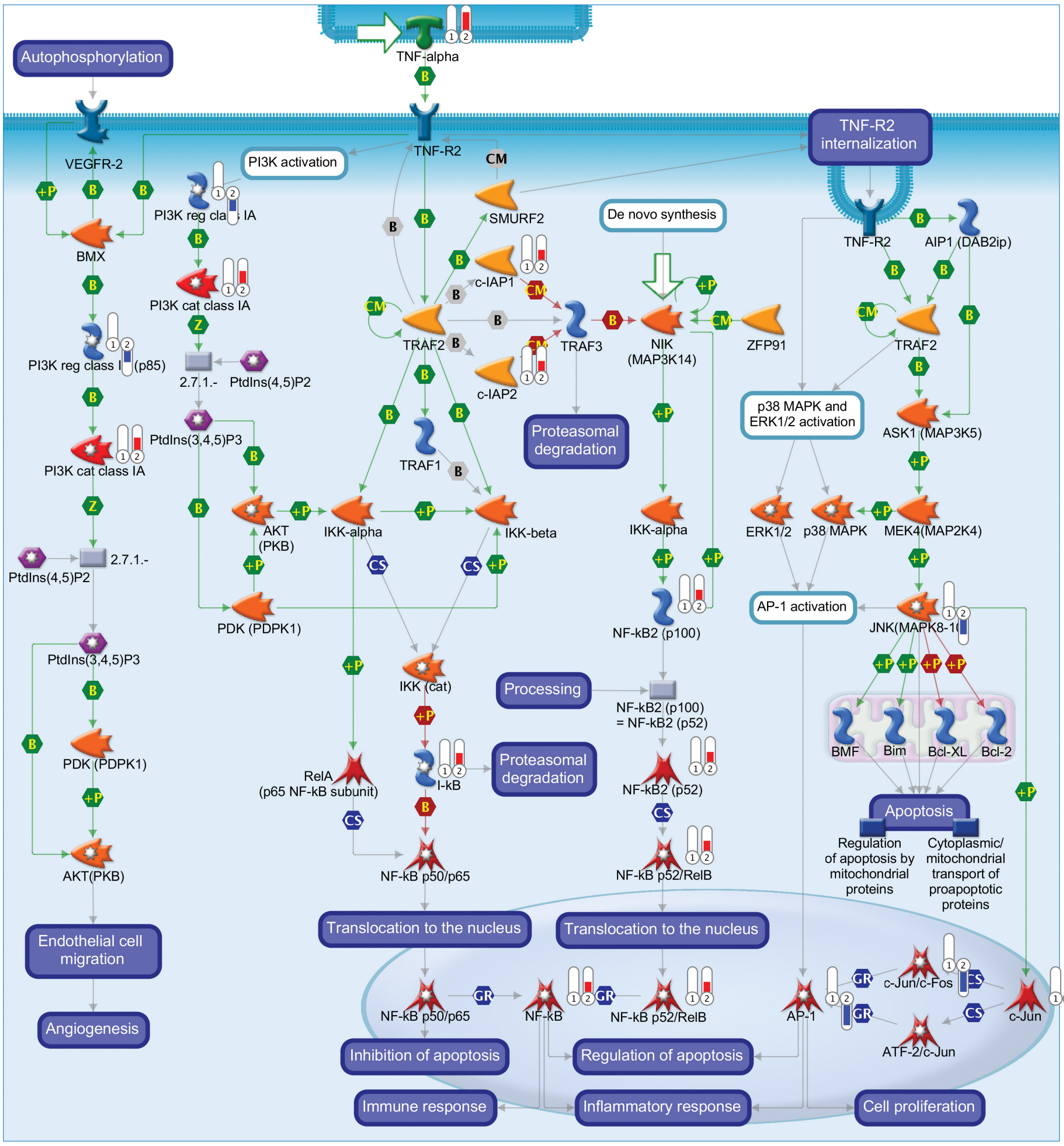

Supplementary Figure S5: ***Metacore analysis of process Immune response- TNF pathway***. Differentially expressed genes are shown with two bars (1 and 2) by their side, where the first bar represents the status of the control set and the second depicts the cumulative status of LNTB patients. If a gene exhibits over expression in patients as compared to control, the second bar (bar 2) contains red fill, and the height of fill denotes extent of overexpression, on the contrary, genes under expressing in patients than controls, contain blue fill in their second bar (bar 2) and height of the fill depicting extent of under regulation. Detailed legend for symbols used in this image can be found at www.pathwaymaps.com/pdf/MC_legend.pdf.

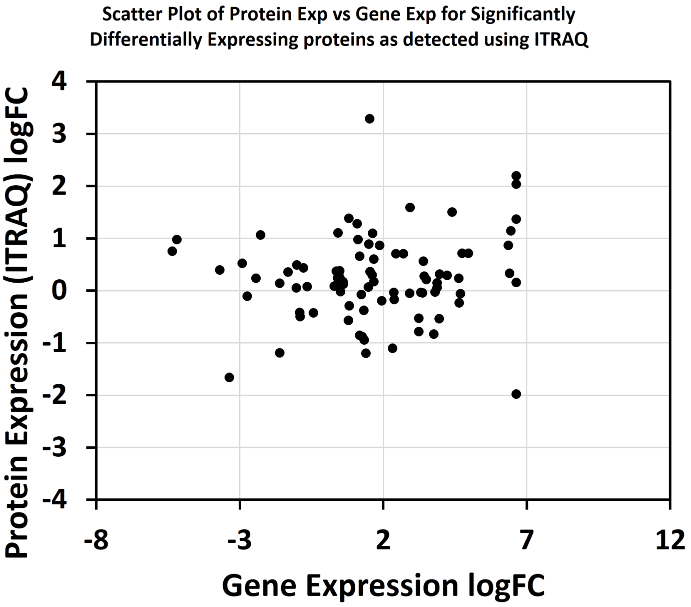

- Pearson's Correlation Test
  - Cor = 0.1257327
- Spearman's Correlation Test
  - Rho = 0.09008865
  - p-value = 0.4094

Supplementary Figure S6: ***Correlation of proteome and transcriptome.*** Scatter plot and correlation analysis of genes and proteins detected in common across global transcriptome and proteome profiling. The log fold change (logFC) values of differential expression of proteins and their corresponding transcripts are shown along the y- and x-axis, respectively.

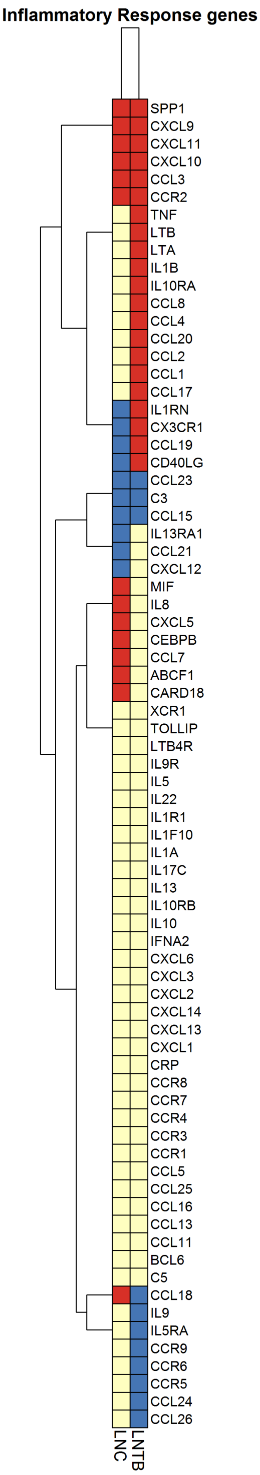

Supplementary Figure S7: ***Qualitative heat map analysis of inflammatory response genes in LNTB and LNC.*** Microarray datasets of current study (LNTB) and LNC (GSE51985) were considered for this analysis. The subsets mentioned in Qiagen SABiosciences online resource. The results presented represent the differential gene expression status in the disease in relation to the controls chosen in respective studies. Unsupervised hierarchical clustering

was performed. Upregulated genes are marked by the red boxes; downregulated genes by blue boxes and yellow boxes indicate lack of differential expression.

(A) (B)

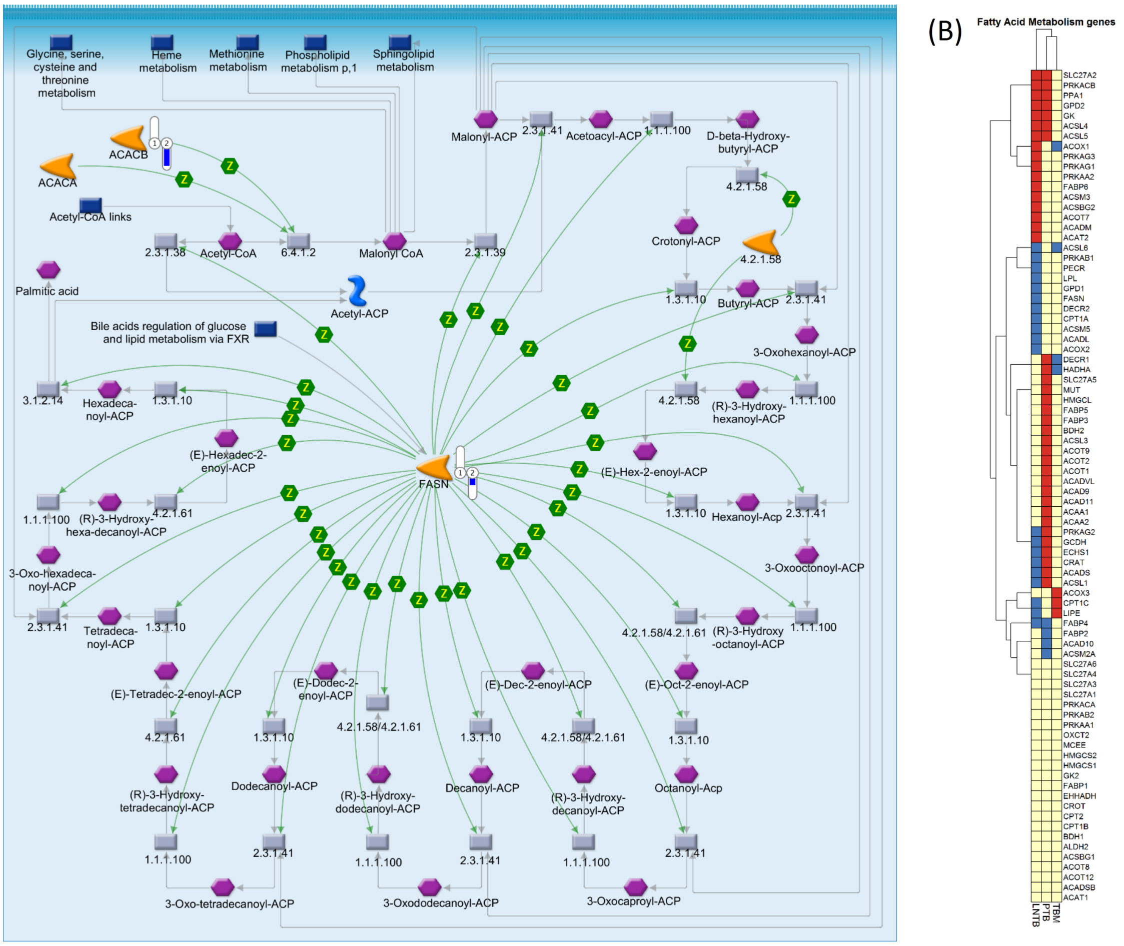

Supplementary Figure S8 (a) ***MetaCore analysis of gene enrichment in Fatty Acid Synthesis pathway.*** Differentially expressed genes are shown with two bars (1 and 2) by their side, where the first bar represents the status of the control set and the second depicts the cumulative status of LNTB patients. If a gene exhibits over expression in patients as compared to control, the second bar (bar 2) contains red fill, and the height of fill denotes extent of overexpression, on the contrary, genes under expressing in patients than controls, contain blue fill in their second bar (bar 2) and height of the fill depicting extent of under regulation. Detailed legend for symbols used in this image can be found at www.pathwaymaps.com/pdf/MC_legend.pdf. **(b)** ***Qualitative heat map analysis of Fatty***

Acid Metabolism genes in LNTB, PTB and TBM. Heat map of comparative expression of genes belonging to fatty acid metabolism. Microarray datasets of current study (LNTB), PTB (GSE20050) and TBM (GSE23074) were considered for this analysis. The results represent the differential gene expression status in the disease in relation to the controls chosen in respective studies. Unsupervised hierarchical clustering was performed. Dendrograms depict unsupervised hierarchical clustering based on their differential expression status. Upregulated genes are marked by red boxes; downregulated genes by blue boxes and yellow boxes indicate lack of differential expression.

Supplementary Tables can be accessed through this link: https://drive.google.com/folderview?id=0B5VkVkkTO_nnVE5kMGxUU1JtNTQ&usp=sharing

Supplementary Table ST1: **List of differentially expressed probes and their annotation obtained by global transcriptome profile of LNTB.** (Provided as Excel file)

Supplementary Table ST2: **DAVID Biological Process annotation of top 10% significantly upregulated and downregulated genes in LNTB and LNC.** (Provided as pdf file)

Supplementary Table ST3: **List of common genes between 393-transcript signature described by Berry et al and LNTB.** (Provided as word file)

Supplementary Table ST4: **iTRAQ Protein Pilot summary analysis file (**Provided as excel file)

Supplementary Table ST5: **List of differentially expressed proteins and their corresponding gene expression status.** (Provided as word file)
